# Supplementary material for: A protein tertiary structure mimetic modulator of the Hippo signalling pathway
Source: Nat Commun. 2020 Oct 27;11:5425. doi: 10.1038/s41467-020-19224-8 (PMC7591920; doi:10.1038/s41467-020-19224-8)
Supplement: Supplementary file 1 — Supplementary Information [file 41467_2020_19224_MOESM1_ESM.pdf]

Supplementary Information

A protein tertiary structure mimetic modulator of the Hippo signalling pathway

Hélène Adihou et al.

1. Supplementary figures

hTEAD1(209-259): RSIGTTKLRLVEFSAFLEQQRDPDSYNKHLFVHIGHANHSYSDPLLESVDI

mTEAD4(209-259): •••ASS••WMl•••••••R•Q•••T•••••••••SQSSP•••••Y••T•••

hTEAD1(260-310): YDKFPEKKGGLKELFGKGPQNAFFLVKFWADLNCNIQDDAGAFYGVTSQYE

mTEAD4(260-310): •••••••••••••••ER••S•••••••••••••T••D•E•••••••S••••

hTEAD1(311-361): SSENMTVTCSTKVCSFGKQVVEKVETEYARFENGRFVYRINRSPM**CEYMIN**

mTEAD4(311-361): •P•••II•••••••••••••••••••••••Y•••HYL•••H•••L••••••

hTEAD1(362-412): **FIHKLKHLPEKYMMNS**VLENFTILLVVTNRDTQETLLCMACVFEVSNSEHG

mTEAD4(362-412): ••••••••••••••••••••••••Q•••••••••••••I•Y•••••A••••

hTEAD1(413-427): AQHHIYRLVKD

mTEAD4(413-427): ••••••••••E

Supplementary Figure 1 | Sequence alignment of hTEAD1 and mTEAD4 Yap binding domain. The residues involved in the binding of two-helix motif of VGL4 are shown in bold.

hVGL4(1- 47): ------METPLDVLSRAASLVHADDEKREAALRGEPRMQTLPVASALSSHRT

mVGL4(1- 52): MLFMKMDLLNYQYLDKMNNNIGVLCYEG••S•••••••••••••••••••••

hVGL4(48- 99): GPPPISPSKRKFSMEPGDEDLDCDNDHVSKMSRIFNPHLNKTANGDCRRDPR

mVGL4(53-104): ••••••••••••••••••K••••E•••••••••••S•••••V••••••••••

hVGL4(100-151): ERSRSPIERAVAPTMSLHGSHLYTSLPSLGLEQPLALTKNSLDASRPAGLSP

mVGL4(105-152): ••••••••••A••AV••••G•••A•••••-M••••••••••S•TG•S•V---

hVGL4(152-203): TLTPGERQQNRPSVITCASAGARNCNLSHCPIAHSGCAAPGPASYRRPPSAA

mVGL4(153-199): -----••••••••••••••••••••••••••••S•••S•••••••••T

hVGL4(204-255): TTC**DPVVEEHFRRSLGKNYKEPEPAPNSVSITGSVDDHFAKALGDTWLQIKA**

mVGL4(200-251): A•••

hVGL4(256-290): **A**KDGASSSPESASRRGQPASPSAHMVSHSHSPSVVS

mVGL4(252-287): •••S•••••••••••••••••T••••••••••••••

Supplementary Figure 2 | Sequence alignment of the hVGL4 and mVGL4. TEAD binding domain is shown in bold.

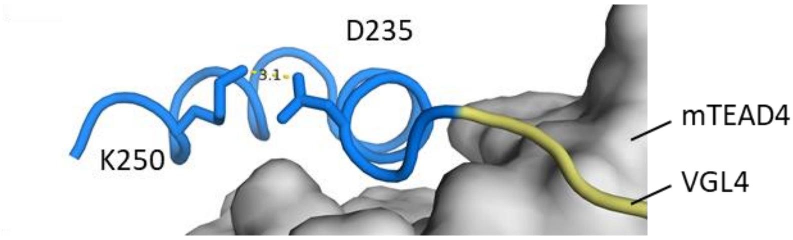

Supplementary Figure 3 | Two-helix motif of VGL4 (blue) bound to mTEAD4 (grey) (PDB ID: 4LN0). The tertiary structure is stabilized by a salt bridge between K235 and D250.

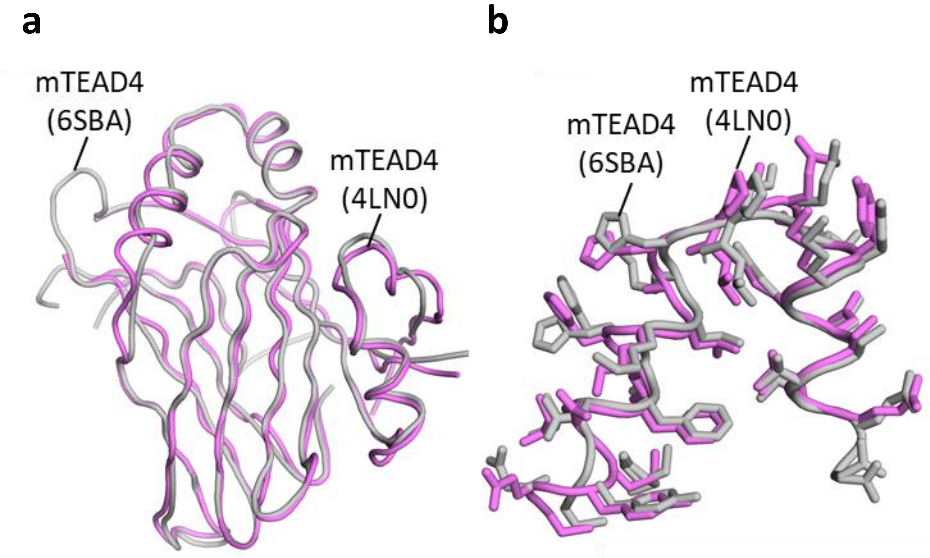

Supplementary Figure 4 | Comparison of crystal structures of VGL4 and **4E** bound to mTEAD4. (a) Superimposition of mTEAD4 bound to **4E** (in grey, PDB ID: 6SBA) and mTEAD4 bond to VGL4 (in pink, PDB ID: 4LN0). (b) Close-up of TEAD interface targeted by the two-helix motif. Backbone conformation and side chain orientations are similar for VGL4- (pink) and **4E**-bound (grey) mTEAD4.

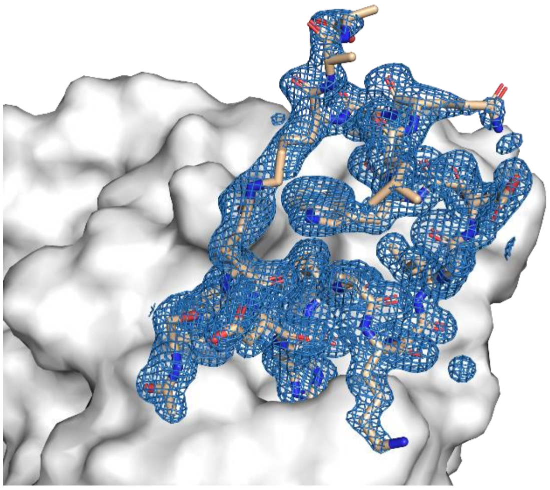

Supplementary Figure 5 | Portion of the 2Fo-Fc electron density map (blue, contoured at σ = 1.2) for the crystal structure of VGL4/**4E**. VGL4 electron density map is represented in grey and **4E** electron density map is represented in blue.

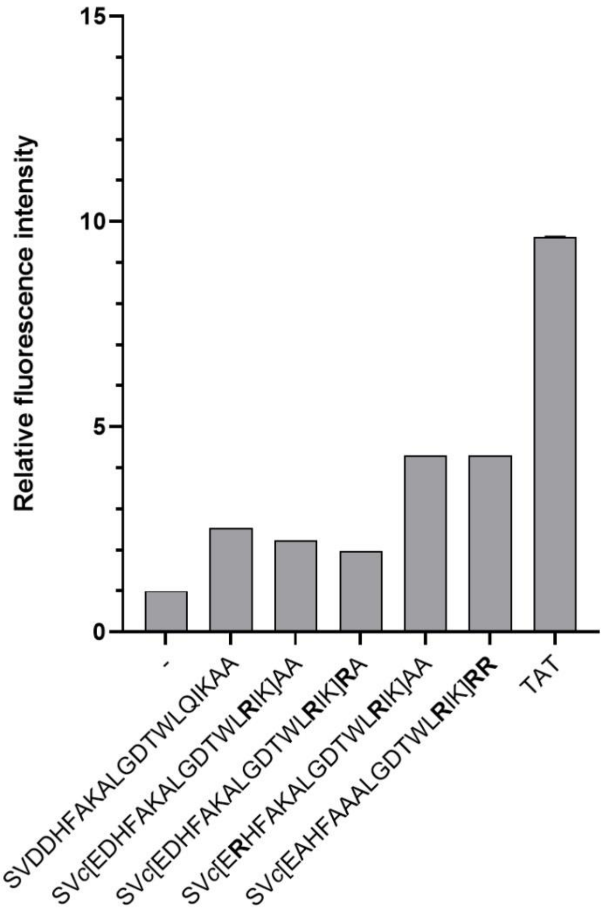

Supplementary Figure 6 | Cell permeability of FITC labelled arginine rich peptides was assessed by flow cytometry in HeLa cells after 90 minutes incubation at 5 µM. The fluorescence intensities are shown relative to DMSO-treated control (-). Source data are provided as a Source Data file.

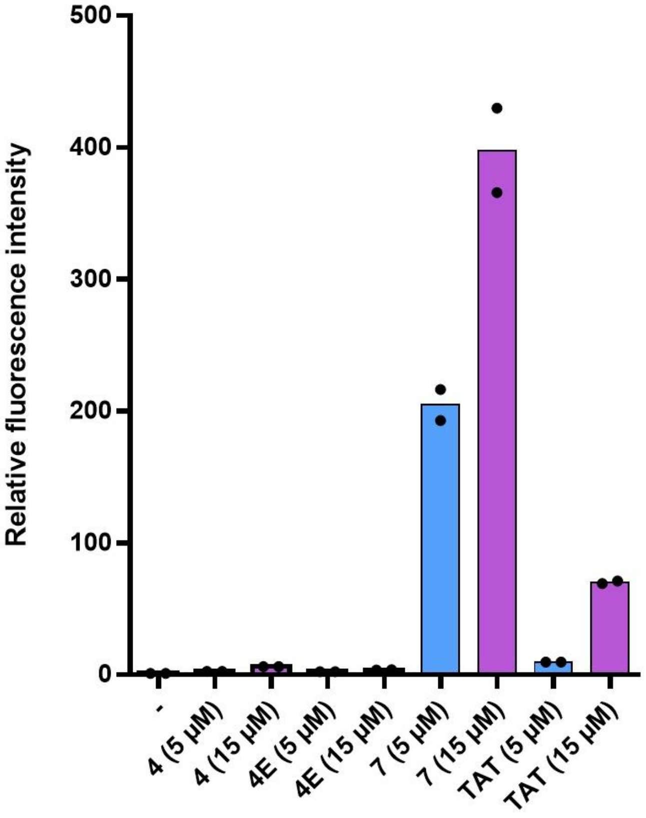

Supplementary Figure 7 | Cell permeability of FITC labelled **4**, **4E**, **7** and Tat was assessed by flow cytometry in HeLa cells after 90 minutes incubation at 5 µM. The fluorescence intensities are shown relative to DMSO-treated control (-) (*n* = 2 biological replicates). Source data are provided as a Source Data file.

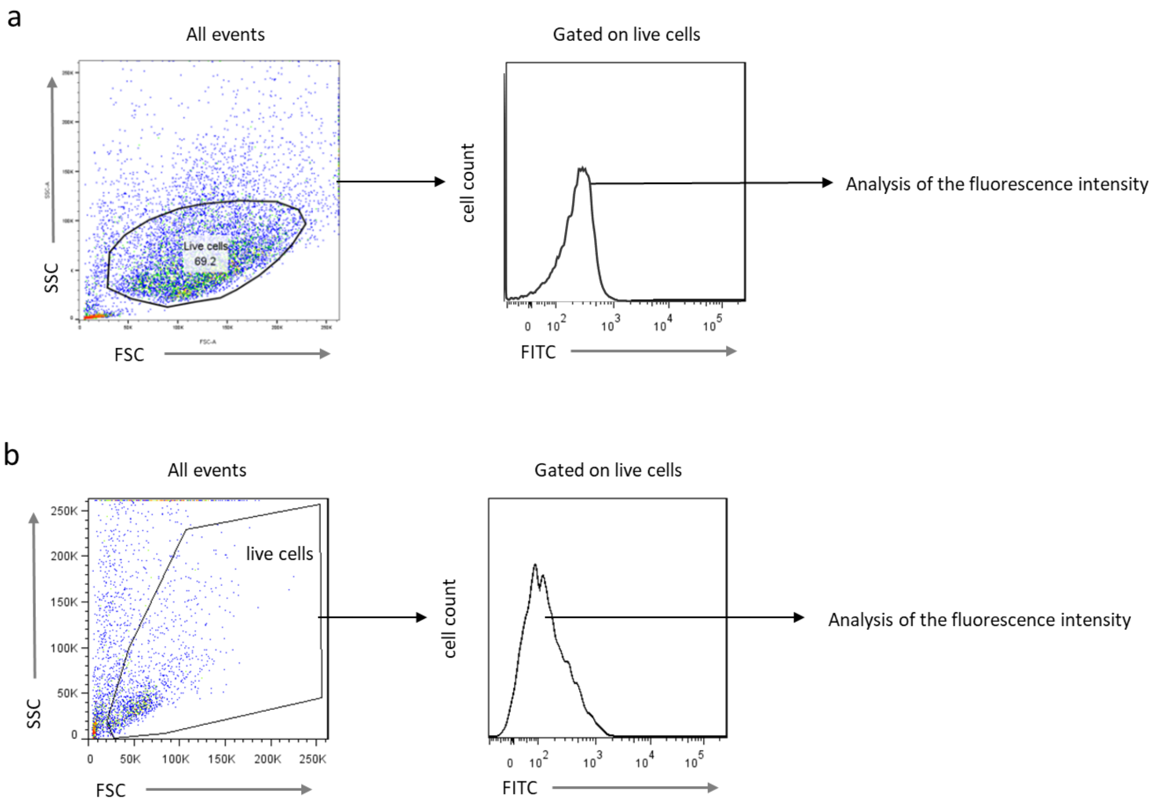

Supplementary Figure 8 | Gating strategies used for cell sorting. (a) Gating strategy to sort HeLa cells for the characterization of the cellular uptake of FITC-labeled peptides presented on Supplementary Figure 5 and 6. (b) Gating strategy to sort whole heart cells for the characterization of the cellular uptake of FITC-labeled peptides presented on Supplementary Figure 16c.

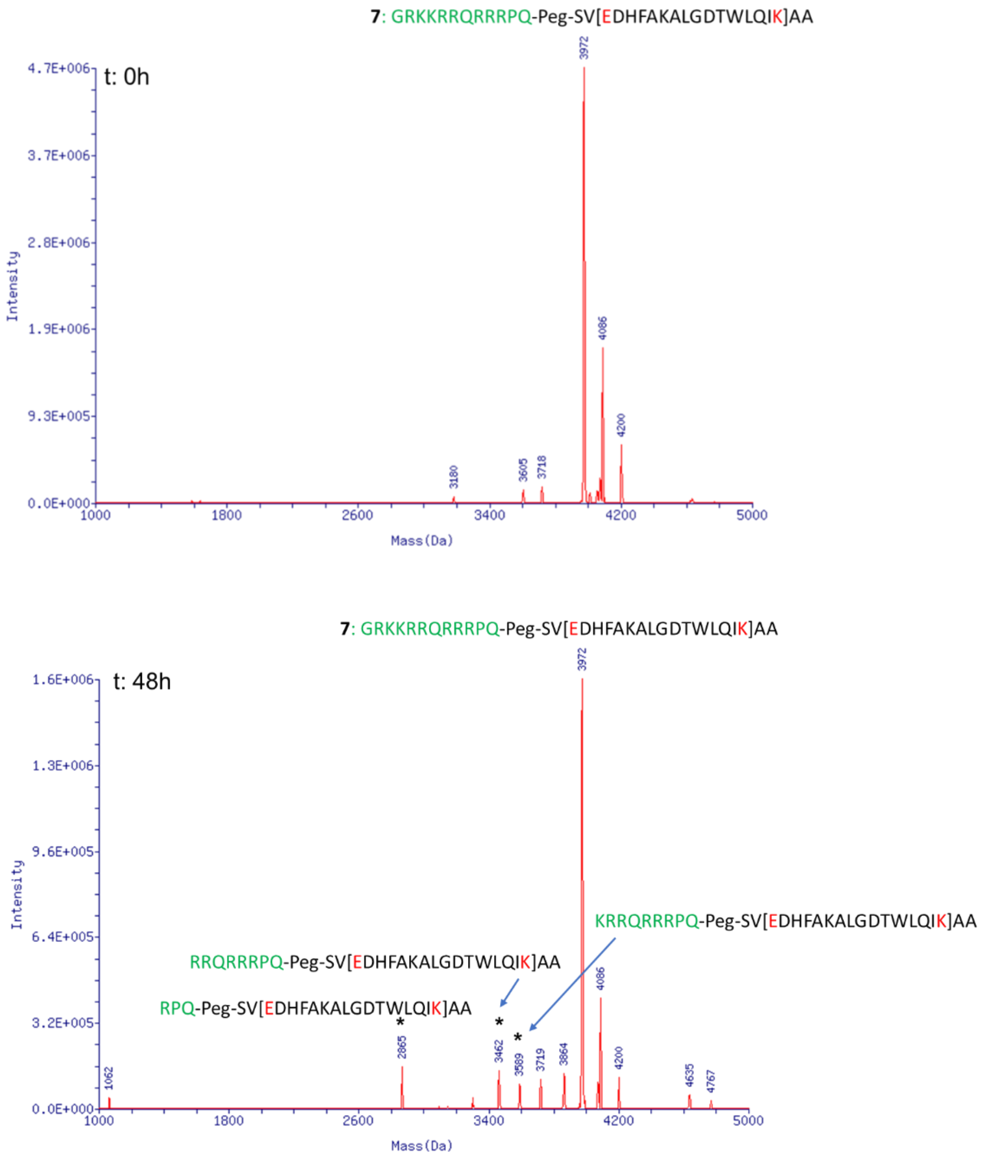

Supplementary Figure 9 │Proteolytic stability of the peptide **7** (30 µM) was evaluated after incubation in cell culture media buffer containing 10% of Fetal Bovine Serum for 48 hours. Deconvolution MS Spectra at 0 h and 48 h were analysed for the identification of the cleavage sites.

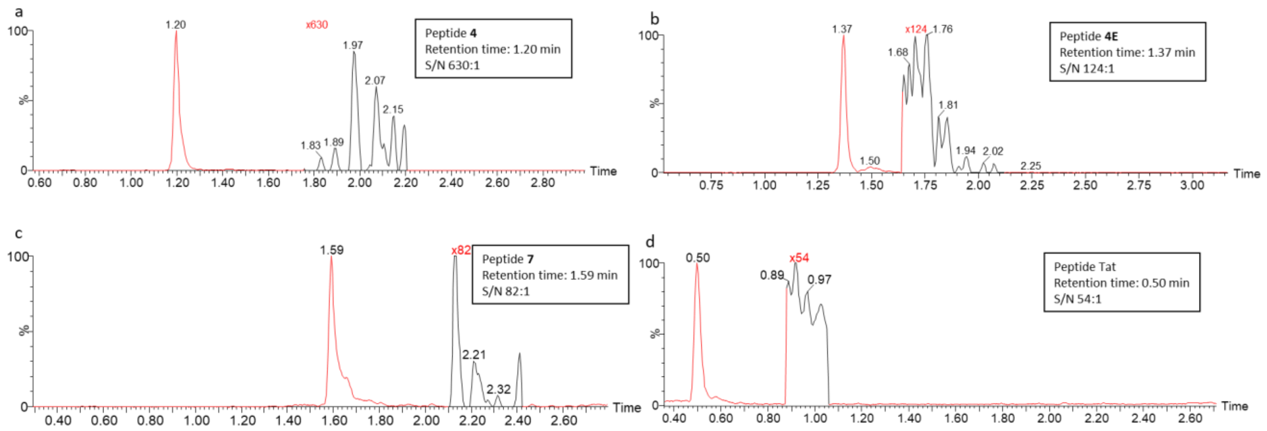

Supplementary Figure 10 | Representative chromatograms used to determine subcellular concentrations of peptides showing S/N ratio at 50 nM of peptide.

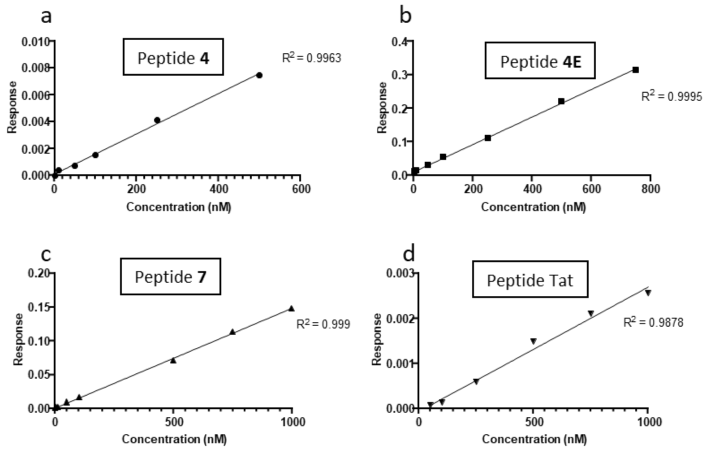

Supplementary Figure 11 | Representative standard calibration curve of peptides (a) **4**, (b) **4E**, (c) **7** and (d) **Tat** used to determine subcellular concentrations of peptides. Source data are provided as a Source Data file.

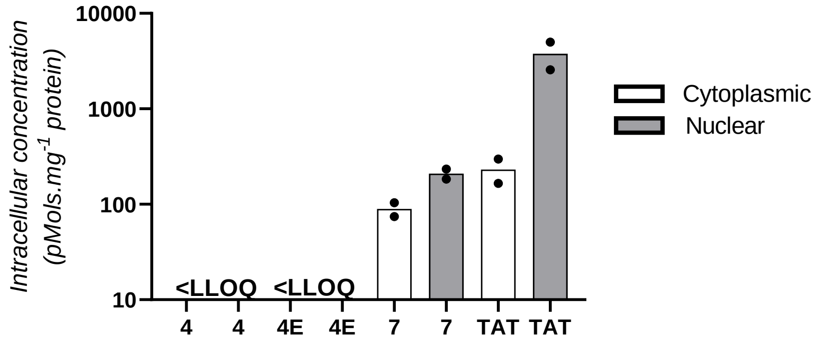

Supplementary Figure 12 | Intracellular concentration of unlabelled peptides investigated by mass spectrometry (24 hours incubation at c(peptide) = 25 µM). Peptide **4** and peptide **4E** have concentrations below the lower limit of quantification (LLOQ), therefore they were not detected. Mean ± SEM were plotted for all data for N=2 biological replicates. Source data are provided as a Source Data file.

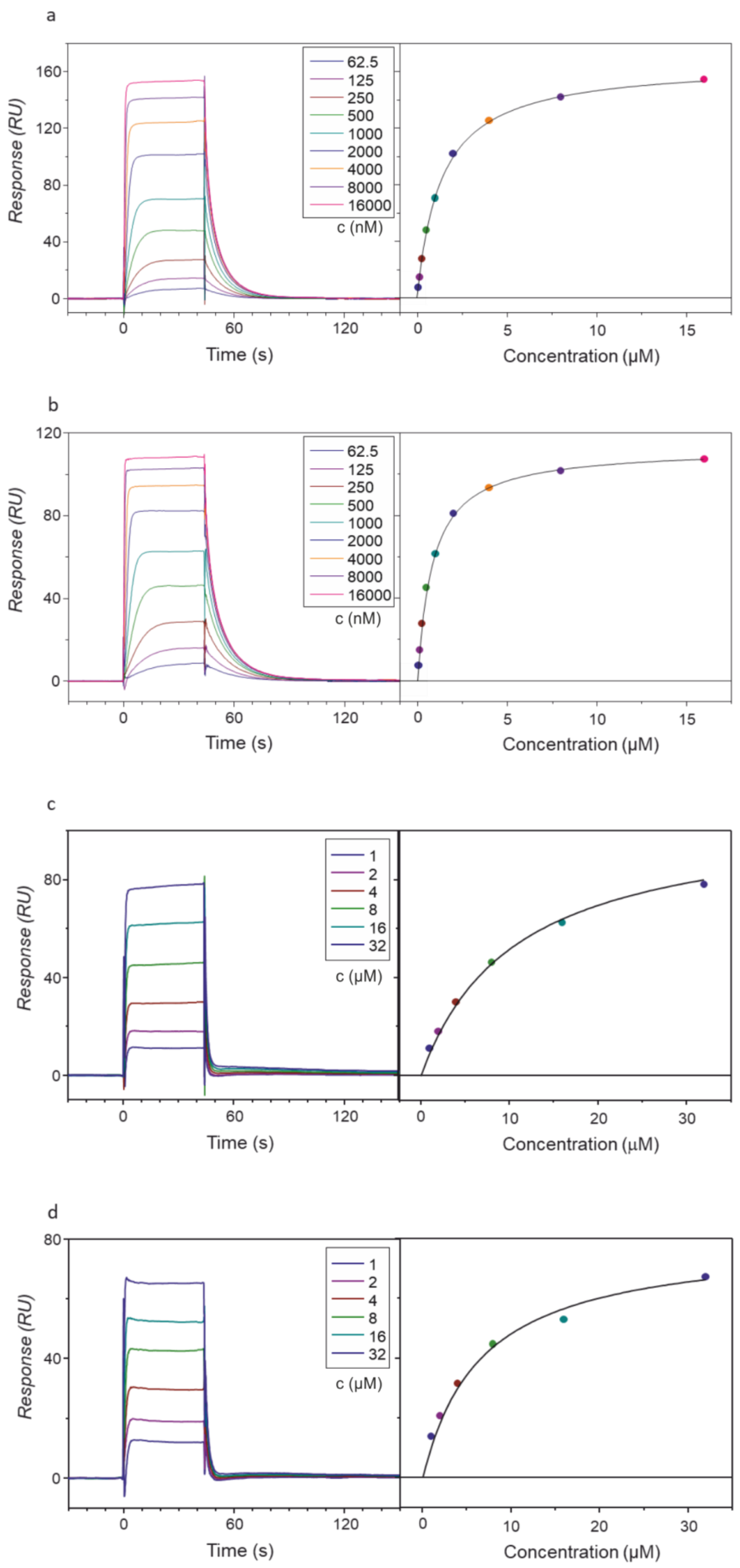

Supplementary Figure 13 | Surface Plasmon Resonance kinetic analyses to measure affinities of peptide **4E** and peptide **4** for hTEAD1 and mTEAD4. SPR assay was performed in HBS-P(+) at 20°C and a flow-rate of 30 µL/min. hTEAD1 has been tethered to a CM5 sensor via amine-coupling using 10 mM MES pH 6.4 during coupling. Representative sensorgrams and saturation binding curves fitted with 1:1 binding model for (a) peptide **4E** to hTEAD1 (K_d= 0.70 ± 0.05 µM), (b) peptide **4E** to mTEAD4 (K_d= 1.2 ± 0.1 µM), (c) peptide **4** to hTEAD1 (K_d= 0.83 ± 0.78 µM), (d) peptide **4** to mTEAD4 (K_d= 1.5 ± 1.0 µM). Source data are provided as a Source Data file.

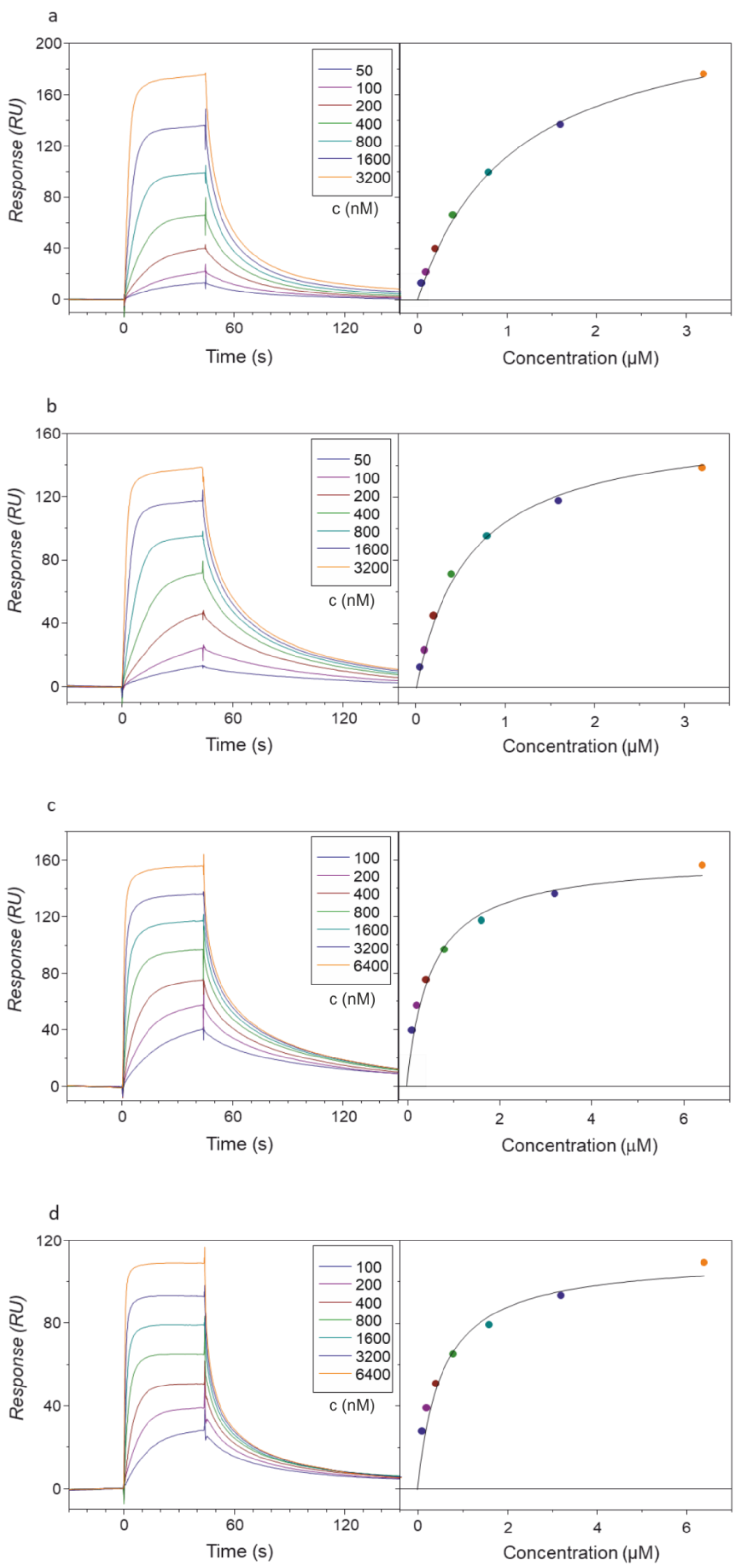

Supplementary Figure 14 | Surface Plasmon Resonance kinetic analyses to measure affinities of YAP and VGL4 for hTEAD1 and mTEAD4. SPR assay was performed in HBS-P(+) at 20°C and a flow-rate of 30 µL/min. hTEAD1 has been tethered to a CM5 sensor via amine-coupling using 10 mM MES pH 6.4 during coupling. Representative sensorgrams and saturation binding curves fitted with steady state affinity model for (a) YAP to hTEAD1(K_d=1.8 µM), (b) YAP to mTEAD4(K_d=0.7 µM), (c) VGL4 to hTEAD1 (K_d=4.3 µM), (d) VGL4 to mTEAD4 (K_d=3.1 µM). Mean ± SEM of triplicates were plotted for all data. Source data are provided as a Source Data file.

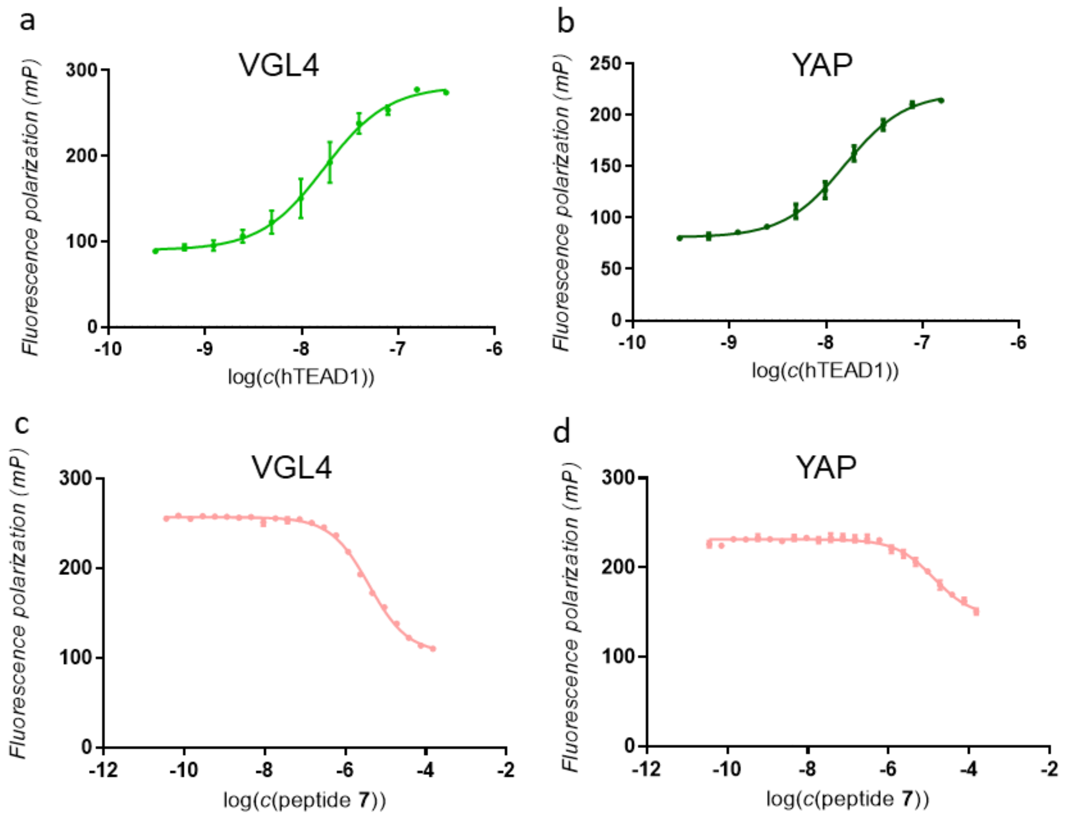

Supplementary Figure 15│ Binding studies of hTEAD1 by fluorescence polarization. Dissociation constants were measured with FITC labelled VGL4 and YAP. The fluorescent cofactor (10 nM) are directly titrated with hTEAD1. After 1 hour of incubation, fluorescence polarization was measured, and a non-linear fitting was applied to the curves to determine the dissociation constants (a) K_d of FITC-VGL4= 17.0 ± 2.5 nM (b) K_d of FITC-YAP = 15.5 ± 1.4 nM. In the competitive binding assay, first a complex with the fluorescent cofactor (10 nM) and hTEAD1 (100 nM) was formed and after 1 hour of incubation, the unlabelled peptide was titrated. After 1 hour of incubation, fluorescence polarization was measured, and a non-linear fitting was applied to the curves to determine the half maximal inhibitory concentration. (c) Competition assay with labelled VGL4 and **7** as competitor (IC_{50} = 5.46 ± 0.25 µM). (d) Competition assay with labelled YAP and **7** as competitor (IC_{50} = 87.7 ± 7.2 µM). Mean ± SEM of triplicates were plotted for all data. Source data are provided as a Source Data file.

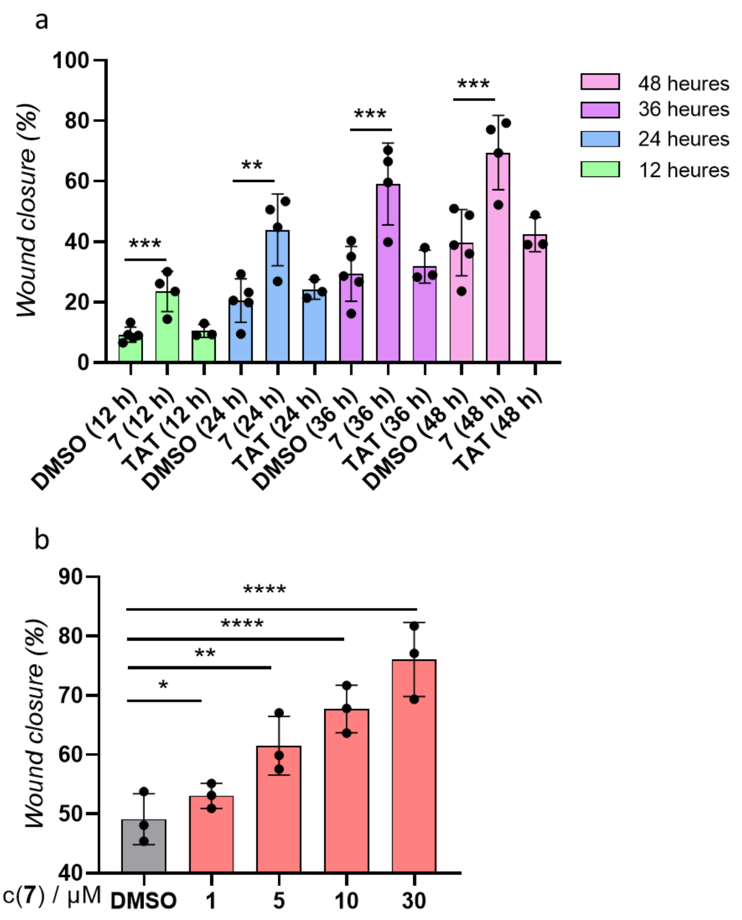

Supplementary Figure 16 | (a) Time-dependent Wound Healing Assay in RKO cells treated with peptide **7**. After the wound formation, RKO cells were incubated with **7** (*c* = 30 μM, cell front after 48 hours of incubation). (b) Dose-dependent Wound Healing Assay with RKO cells treated with peptide **7**. After the wound formation, RKO cells were incubated with **7** (*c* = 1, 5, 10 and 30 μM) for 48 h of incubation. Number of independent biological replicates: n = 3, error bars = SEM. For all the experiments, statistical significances were determined by Student's paired t test. P<0.05 was considered statistically significant ($p < 0.05$ = *; p< 0,01 = **; $p < 0.001$ = ***, $p < 0.0001$ = ****) and source data are provided as a Source Data file.

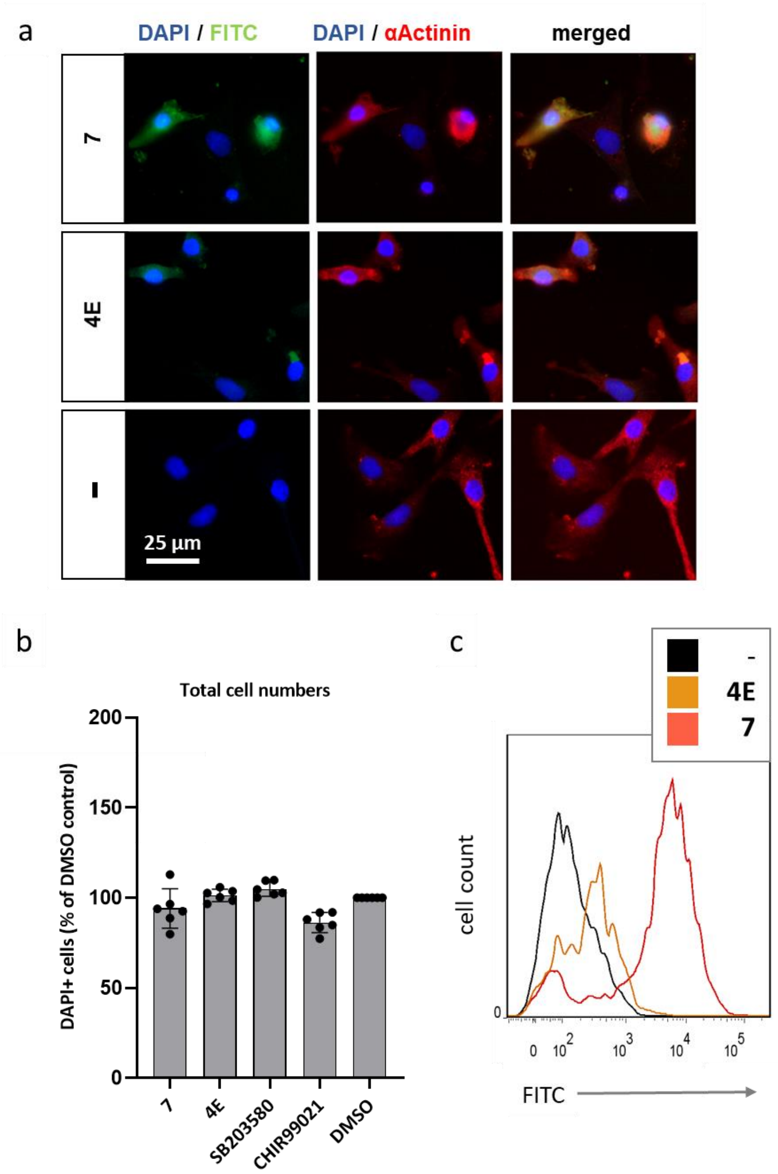

Supplementary Figure 17 | (a) Representative micrographs supporting the cellular uptake of FITC-labelled **7** and **4E** in whole heart cells monitored by fluorescence microscopy after 90 minutes treatments (scale bar: 25 µm), number of technical replicates: N = 3, number of independent biological replicates: n = 1; (b) Cell toxicity assessment of peptides **7** and **4E** as well as control compounds (SB203580, CHIR99021) on rat heart cells in culture (*c*(peptide) = 10 µM). Number of independent biological replicates: n = 6, error bars = SEM. (c) Cellular uptake of FITC-labelled peptides **7** and **4E** by flow cytometry analysis of live cells after 90 min incubation at 5 µM: Representative histogram plot, (-) DMSO control). Source data are provided as a Source Data file.

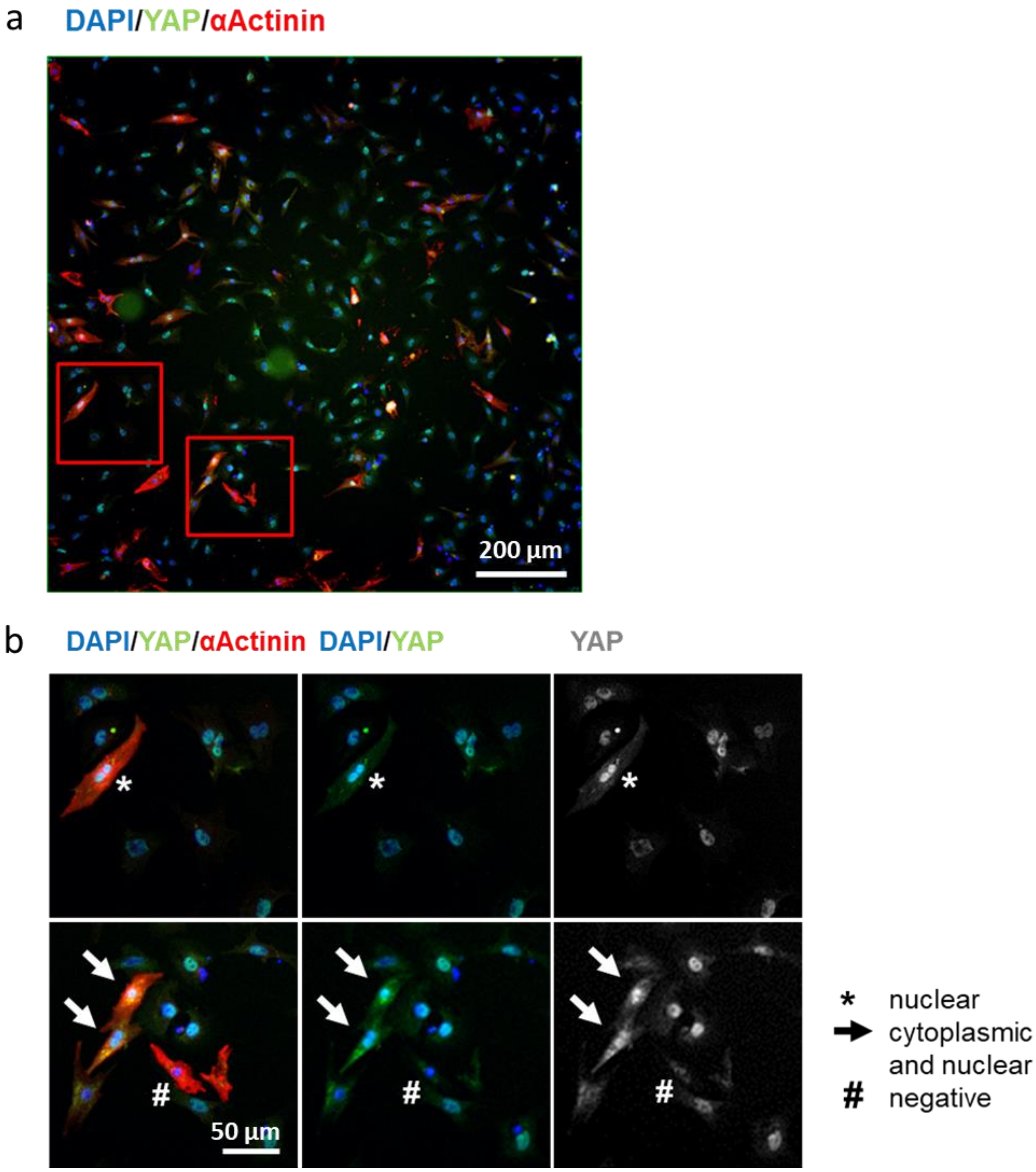

Supplementary Figure 18 | Analysis of sub-cellular distribution of YAP in cardiomyocytes after treatment.
(a) Representative fluorescence images showing the different localization of YAP (green) in cardiomyocytes (red), .Scale bar: 200 µm (lower magnification photography). (b) Optical section of the red rectangles with (*) labels a cardiomyocyte showing nuclear YAP staining, (→) label cardiomyocytes showing YAP localized in the nucleus and in the cytoplasm, and (#) labels a cardiomyocyte negative for YAP staining. Scale bar = 50 µm. Number of independent biological replicates: n = 2.

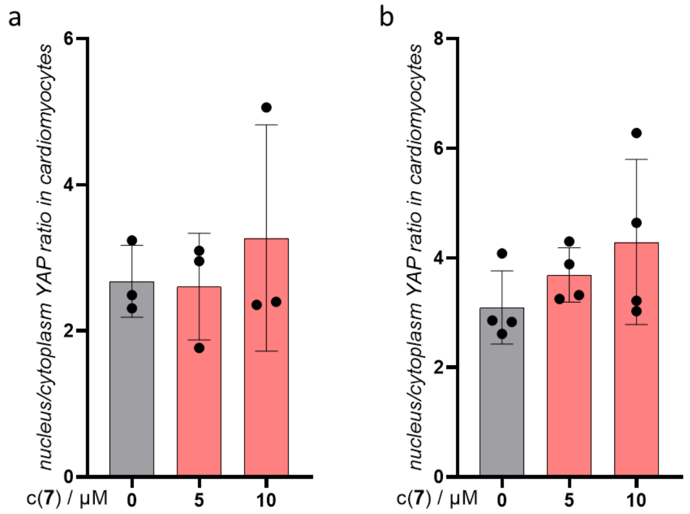

Supplementary Figure 19 | Analysis of sub-cellular distribution of YAP in cardiomyocytes after treatment. The graphs depict nuclear to cytoplasmic YAP ratio in cardiomyocytes (a) 8 hours and (b) 24 h after treatment. YAP is detected by fluorescence immunocytochemistry. Fluorescence intensity is quantified in the specific cell compartments and analyzed (*n* = 3-4 biologically independent replicates; error bars represent SEM). Source data are provided as a Source Data file.

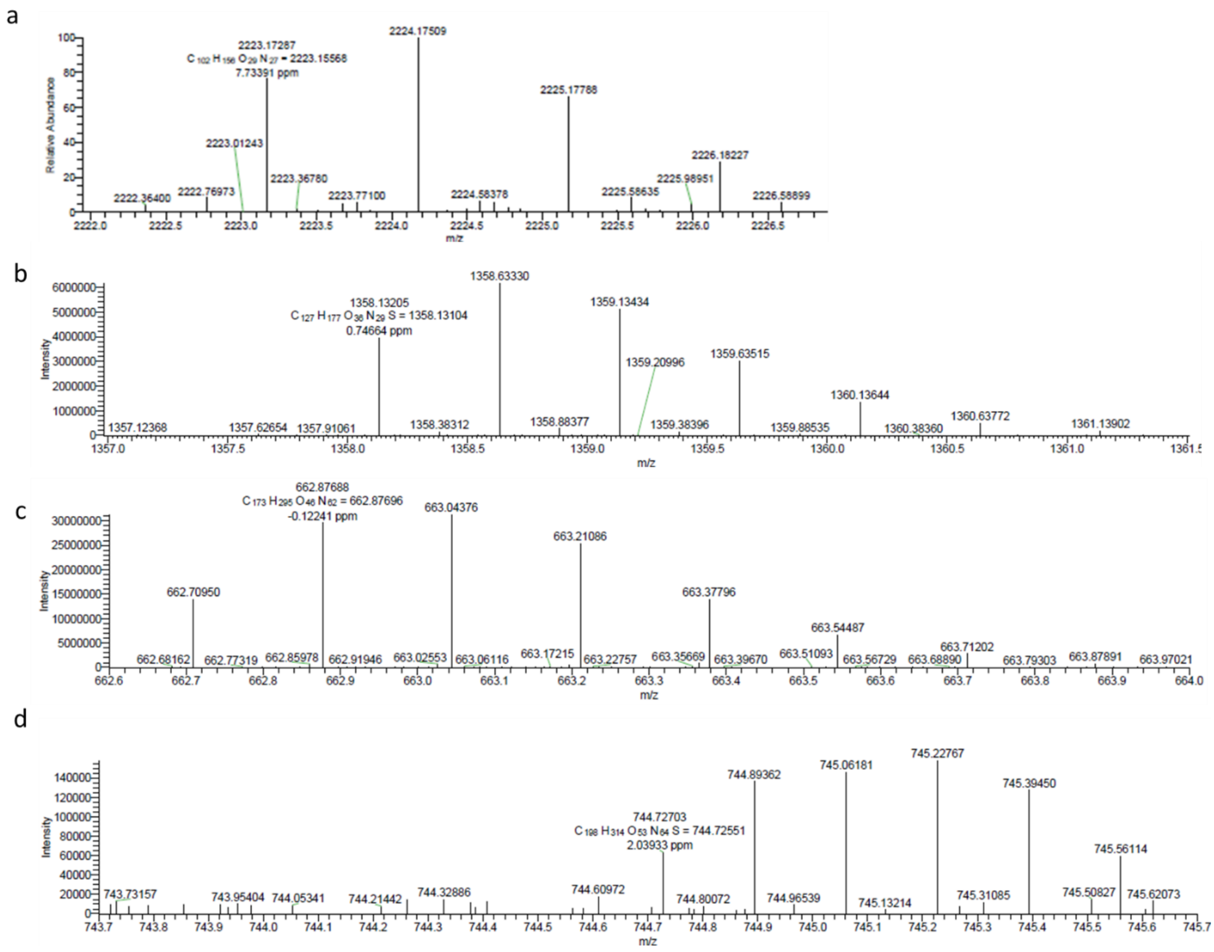

Supplementary Figure 20 | Analytical characterization of peptide **4E** and peptide **7**. (a) High resolution mass spectrometry of acetylated peptide **4E**. HRMS (m/z): $[M+H]^+$ calcd. for $C_{102}H_{156}N_{29}O_{27}$, 2223.1557; found, 2223.1729. (b) High resolution mass spectrometry of FITC labelled peptide **4E**. HRMS (m/z): $[M+2H]^{2+}$ calcd. for $C_{127}H_{177}N_{36}O_{29}S$, 1358.1310; found, 1358.1320. (c) High resolution mass spectrometry of acetylated peptide **7**. HRMS (m/z): $[M+6H]^{6+}$ calcd. for $C_{173}H_{295}N_{46}O_{62}$, 662.8769; found, 662.8769. (d) High resolution mass spectrometry of FITC labelled peptide **7**. HRMS (m/z): $[M+6H]^{6+}$ calcd. for $C_{198}H_{314}N_{53}O_{64}S$, 744.7255; found, 744.7270.

2. Supplementary tables

Supplementary Table 1: Sequences of the linear peptide precursors including K_d-values as obtained by SPR with either mTEAD4 or hTEAD1.

	**Amino acid sequence**	**mTEAD4** K_d/ µM	**hTEAD1** K_d/ µM
**4**	SVDDHFAKALGDTWLQIKAA	3.1	4.3
**4E(open)**	SVEDHFAKALGDTWLQIKAA	2.4	3.1
**9**	SVDDHFAKALGDTWLQIOAA	3.2	4. 1
**10**	SVEDHFAKALGDTWLQIOAA	4.2	5.7
**11**	SVDDHFAKALGDTWLQIBAA	5.3	5.5
**12**	SVEDHFAKALGDTWLQIBAA	1.4	1.7
**13**	SVDDHFAKALGDTWLQIAAA	12	17

Supplementary Table 2: Crystallographic data collection and refinement statistics for complex of 4E and mTEAD4 (PDB ID: 6SBA) [a]Values in parentheses represent the highest resolution shell.

**Data collection**	
Space group	P $2_12_12_1$
Cell dimensions	
a, *b*, *c* (Å)	56.5, 65.11, 74.91
α, β, γ (°)	90.00, 90.00, 90.00
Resolution (Å)	49.14-1.30 (1.33-1.30)[a]
R_{sym} or R_{merge}	9.8 (305.7)
$I / \sigma I$	11.96 (0.85)
Completeness (%)	97.9 (96.1)
Redundancy	13.3 (13.04)
**Refinement**	
Resolution (Å)	49.1-1.34
No. reflections	67170
R_{work} / R_{free}	15.1/17.5
No. atoms	
Protein	2073
Ligand/ion	115
Water	134
B-factors	
Protein	36.84
Ligand/ion	
Water	47.5
R.m.s. deviations	
Bond lengths (Å)	0.024
Bond angles (°)	2.028

Supplementary Table 3: Alanine scan, K_d-values were determined by SPR using either mTEAD4 or hTEAD1. In addition, IC_{50}-values based on competition fluorescence polarization are given.

	Amino acid sequence	hTEAD1 K_d/ µM	mTEAD4 K_d/ µM	mTEAD4 IC_{50}/ µM
**14**	AV*c*[EDHFAKALGDTWLQIK]AA	1.5 ± 0.020	2.7 ± 0.25	6.1 ± 4.3
**15**	SA*c*[EDHFAKALGDTWLQIK]AA	1.0 ± 0.37	1.5 ± 0.37	2.1 ± 0.56
**16**	SV*c*[EAHFAKALGDTWLQIK]AA	0.74 ± 0.46	1.6 ± 0.69	3.0 ± 0.76
**17**	SV*c*[EDAFAKALGDTWLQIK]AA	>100	>100	>100
**18**	SV*c*[EDHAAKALGDTWLQIK]AA	>100	>100	>50
**19**	SV*c*[EDHFAAALGDTWLQIK]AA	1.7 ± 0.010	2.8 ± 0.060	0.28 ± 0.090
**20**	SV*c*[EDHFAKAAGDTWLQIK]AA	>100	>100	>100
**21**	SV*c*[EDHFAKALGATWLQIK]AA	>100	>100	>100
**22**	SV*c*[EDHFAKALGDAWLQIK]AA	91 ± 14	113 ± 3.5	>100
**23**	SV*c*[EDHFAKALGDTALQIK]AA	7.8 ± 0.15	15± 0.85	>50
**24**	SV*c*[EDHFAKALGDTWAQIK]AA	1.1 ± 0.06	2.0 ± 0.14	0.10 ± 0.01
**25**	SV*c*[EDHFAKALGDTWLAIK]AA	0.75 ± 0.12	1.3 ± 0.11	0.060 ± 0.010
**26**	SV*c*[EDHFAKALGDTWLQAK]AA	2.7 ± 1.07	4.4 ± 1.35	7.4 ± 6.1

Supplementary Table 4: Sequences and overall charges of the peptides to investigate the influence of arginine content in cellular uptake.

	Amino acid sequence	Charge
**4**	SVDDHFAKALGDTWLQIKAA	-1
**4E**	SV*c*[EDHFAKALGDTWLQIK]AA	-1
**27**	SV*c*[EDHFAKALGDTWLRIK]AA	0
**28**	SV*c*[EDHFAKALGDTWLRIK]RA	+1
**29**	SV*c*[EAHFAAALGDTWLRIK]RR	+2
**7**	GRKKRRQRRRPQ-Peg-SV*c*[EDHFAKALGDTWLQIK]AA	+7
**Tat**	GRKKRRQRRRPQ	+8

Supplementary Table 5: Dissociation constant (K_d) of peptides **4E** and **7** evaluated by SPR and FP

	Amino acid sequence	SPR		FP	
		**hTEAD1 K_d/ µM**	**mTEAD4 K_d/ µM**	**hTEAD1 K_d/ µM**	**mTEAD4 K_d/ µM**
**4E**	SV[EDHFAKALGDTWLQIK]AA	0.70 ± 0.05	1.2 ± 0.4	0.168 ± 0.037	0.032 ± 0.005
**7**	GRKKRRQRRRPQ-Peg-SV[EDHFAKALGDTWLQIK]AA	0.83 ± 0.78	1.5 ± 1.0	0.147 ± 0.07	0.051 ± 0.005

Supplementary Table 6: Dissociation constant (K_d) of VGL4(203 – 256) and YAP(50 – 100) evaluated by SPR and FP

**Cofactor**	**SPR**		**FP**	
	**hTEAD1 K_d/ uM**	**mTEAD4 K_d/ uM**	**hTEAD1 K_d/ nM**	**mTEAD4 K_d/ nM**
VGL4	1.4	2.0	17.0 ± 2.5	9.2 ± 0.6
YAP	1.8	0.7	15.5 ± 1.4	6.4 ± 0.4

Supplementary Table 7: IC_{50}-values for the inhibition of complex between hTEAD1 and a cofactor with peptide **7** determined by competitive fluorescence polarisation assay.

	**VGL4 IC_{50}/ µM**	**YAP IC_{50}/ µM**
Peptide **7**	5.465 ± 0.253	87.7 ± 7.241

Supplementary Table 8: MS settings for peptide and standard quantification.

**Compound**	**Mode**	**Parent ion**	**Daughter ion**	**Charge state**	**Dwell**	**Cone Voltage**	**Collision Energy**
Peptide **4**	ESI +ve	743.0	83.6	3H+	0.162	30	35
Peptide **7**	ESI +ve	663.0	110.0	6H+	0.162	30	35
Peptide **4E**	ESI +ve	1112.0	159.0	2H+	0.162	35	55
Tat	ESI +ve	333.4	70.0	5H+	0.162	30	35
Clozapine	ESI +ve	327.1	270.1	1H+	0.162	30	35

Supplementary Table 9: Media concentrations pre incubation

**Compound**	**Media Concentration pre incubation / µM**
Peptide **4**	11.5
Peptide **7**	22.4
Peptide **4E**	19.0
Tat	22.4

Supplementary Table 10: Total drug content in RKO cells on incubation of 25 µM compound for 90 minutes.

Compound	Measured Concentration / nM	Dilution Factor	nMol	pMol.mg^{-1}
Peptide **4**	1133.521 ± 133.393	10	1.7002815 ± 0.2000895	1685.1 ± 246.5
Peptide **7**	122.09 ± 7.277	10	0.183135 ± 0.010915	183 ± 53.3
Peptide **4E**	17.2065 ± 0.8045	10	0.02580975 ± 0.0109155	424.4 ± 33.7
Tat	478.752 ± 163.021	10	0.718128 ± 0.010915	882.5 ± 319.5

Supplementary Table 11: Total drug content in RKO cells on incubation of 25 µM compound for 24 hours. (LLOQ: lower limit of quantification)

Compound	Measured Concentration / nM	Dilution Factor	nMol	pMol.mg^{-1}
Peptide **4**	322.145 ± 33.657	10	0.4832175 ± 0.0504855	331.25 ± 86.85
Peptide **7**	91.646 ± 61.816	10	0.137469 ± 0.092724	127.1 ± 102.1
Peptide **4E**	<LLOQ	10	<LLOQ	<LLOQ
Tat	1797.357 ± 596.21	10	2.6960355 ± 0.894315	1647 ± 647

Supplementary Table 12: Sub-cellular drug content in RKO cells on incubation of 25 µM compound for 90 minutes. (LLOQ: lower limit of quantification)

Compound	Sub-cellular compartment	Measured concentration / nM	Dilution Factor	nMol	pMol.mg^{-1}
Peptide **4**	Cytoplasm	<LLOQ	10	<LLOQ	<LLOQ
	Nuclear	<LLOQ	10	<LLOQ	<LLOQ
Peptide **7**	Cytoplasm	265.5865 ± 102.9805	2	0.07967595 ± 0.030894	89.2 ± 14.9
	Nuclear	123.5385 ± 6.5745	10	0.18530775 ± 0.009862	231.15 ± 65.95
Peptide **4E**	Cytoplasm	<LLOQ	10	<LLOQ	<LLOQ
	Nuclear	<LLOQ	10	<LLOQ	<LLOQ
Tat	Cytoplasm	91.363 ± 8.748	10	0.1370445 ± 0.013122	209 ± 25
	Nuclear	416.901 ± 142.802	10	0.6253515 ± 0.625352	3777.5 ± 1210.5

Supplementary Table 13: Sub-cellular drug content in RKO cells on incubation of 25 µM compound for 24 hours. (LLOQ: lower limit of quantification)

Compound	Sub-cellular compartment	Measured concentration / nM	Dilution Factor	nMol	pMol.mg^{-1}
Peptide **4**	Cytoplasm	<LLOQ	10	<LLOQ	<LLOQ
	Nuclear	<LLOQ	10	<LLOQ	<LLOQ
Peptide **7**	Cytoplasm	155.914 ± 40.331	2	0.0467742 ± 0.0120993	48.75 ± 24.55
	Nuclear	202.161 ± 23.082	2	0.0606483 ± 0.0069246	243.25 ± 93.25
Peptide **4E**	Cytoplasm	<LLOQ	10	<LLOQ	<LLOQ
	Nuclear	<LLOQ	10	<LLOQ	<LLOQ
Tat	Cytoplasm	98.42 ± 12.91	10	0.14763 ± 0.01936	109 ± 7
	Nuclear	656.841 ± 162.534	10	0.9852615 ± 0.243801	2890.5 ± 521.5

Supplementary Table 14: r^2 and QC errors

	r^2	QC 500 nM	QC 100 nM
Peptide **4**	0.996	19.5%	9.1%
Peptide **7**	0.999	2.0%	10.9%
Peptide **4E**	0.996	14.0%	18.4%
Tat	0.988	7.8%	17.5%

Supplementary Table 15: relative target quantity (RQ) minimum and maximum by RT-qPCR

Target gene	Compound	RQ	RQ Min	RQ Max
CYR61	Tat	1	0.977	1.024
	Peptide **7**	3.964	3.839	4.094
CTGF	Tat	1	0.895	1.118
	Peptide **7**	1.987	1.843	2.144
ANKRD1	Tat	1	0.893	1.120
	Peptide **7**	4.731	4.459	5.020
SERPINE1	Tat	1	0.977	1.023
	Peptide **7**	3.520	3.277	3.782
CCNA2	Tat	1	0.934	1.071
	Peptide **7**	0.934	0.810	1.078

Supplementary Table 16: Overview of the synthesized peptides. Peptide analysis by mass spectrometry.

	Amino acid sequence	N-terminus	Obs. MS	Calc. MS
**1**	hVGL4(203-256)	Ac	1988.028	1988.010 $[M+3H]^{3+}$
b-**1**	hVGL4(203-256)	Biotin-Peg	1573.559	1573.544 $[M+4H]^{4+}$
**2**	DPVVEEHFRRSLGKNY	Ac	994.013	994.010 $[M+2H]^{2+}$
**3**	KEPEPAPNSVSITG	Ac	1466.757	1466.748 $[M+H]^{+}$
**4**	SVDDHFAKALGDTWLQIKAA	Ac	1114.082	1114.079 $[M+2H]^{2+}$
		FITC-Peg	1360.634	1360.632 $[M+2H]^{2+}$
**4A**	SV*c*[DDHFAKALGDTWLQIB]AA	Ac	1091.058	1091.058 $[M+2H]^{2+}$
**4B**	SV*c*[DDHFAKALGDTWLQIO]AA	Ac	1098.061	1098.066 $[M+2H]^{2+}$
**4C**	SV*c*[DDHFAKALGDTWLQIK]AA	Ac	1105.074	1105.074 $[M+2H]^{2+}$
**4D**	SV*c*[EDHFAKALGDTWLQIO]AA	Ac	1105.582	1105.577 $[M+2H]^{2+}$
**4E**	SV*c*[EDHFAKALGDTWLQIK]AA	Ac	1112.093	1112.081 $[M+2H]^{2+}$
		FITC-Peg	1358.132	1358.131 $[M+2H]^{2+}$
**5**	SVDDHFAKALG	Ac	1200.600	1200.606 $[M+H]^{1+}$
**6**	DTWLQIKAA	Ac	1086.597	1086.594 $[M+2H]^{2+}$
**7**	GRKKRRQRRRPQ-Peg-SV*c*[EDHFAKALGDTWLQIK]AA	Ac	662.877	662.877 $[M+6H]^{6+}$
		FITC-Peg	744.725	744.727 $[M+3H]^{3+}$
**8**	GRKKRRQRRRPQ	Ac	832.026	832.019 $[M+2H]^{2+}$
		FITC-Peg	1078.078	1078.069 $[M+2H]^{2+}$
**4E(open)**	SVEDHFAKALGDTWLQIKAA	Ac	1119.087	1119.593 $[M+2H]^{2+}$
**9**	SVDDHFAKALGDTWLQIOAA	Ac	1106.562	1106.567 $[M+2H]^{2+}$
**10**	SVEDHFAKALGDTWLQIOAA	Ac	1113.570	1113.575 $[M+2H]^{2+}$
**11**	SVDDHFAKALGDTWLQIBAA	Ac	1100.064	1100.064 $[M+2H]^{2+}$
**12**	SVEDHFAKALGDTWLQIBAA	Ac	1106.562	1106.567 $[M+2H]^{2+}$
**13**	SVDDHFAKALGDTWLQIAAA	Ac	1085.551	1085.550 $[M+2H]^{2+}$
**14**	AV*c*[EDHFAKALGDTWLQIK]AA	Ac	1104.096	1104.084 $[M+2H]^{2+}$
**15**	SA*c*[EDHFAKALGDTWLQIK]AA	Ac	1098.077	1098.065 $[M+2H]^{2+}$
**16**	SV*c*[EAHFAKALGDTWLQIK]AA	Ac	1090.098	1090.086 $[M+2H]^{2+}$
**17**	SV*c*[EDAFAKALGDTWLQIK]AA	Ac	2157.153	2157.133 $[M+H]^{+}$
**18**	SV*c*[EDHAAKALGDTWLQIK]AA	Ac	1074.075	1074.065 $[M+2H]^{2+}$
**19**	SV*c*[EDHFAAALGDTWLQIK]AA	Ac	1083.561	1083.552 $[M+2H]^{2+}$
**20**	SV*c*[EDHFAKAAGDTWLQIK]AA	Ac	1091.066	1091.058 $[M+2H]^{2+}$
**21**	SV*c*[EDHFAKALGATWLQIK]AA	Ac	1090.090	1090.086 $[M+2H]^{2+}$
**22**	SV*c*[EDHFAKALGDAWLQIK]AA	Ac	1097.079	1097.076 $[M+2H]^{2+}$
**23**	SV*c*[EDHFAKALGDTALQIK]AA	Ac	1054.564	1054.561 $[M+2H]^{2+}$
**24**	SV*c*[EDHFAKALGDTWAQIK]AA	Ac	1091.061	1091.058 $[M+2H]^{2+}$
**25**	SV*c*[EDHFAKALGDTWLAIK]AA	Ac	1093.059	1093.056 $[M+2H]^{2+}$
**26**	SV*c*[EDHFAKALGDTWLQAK]AA	Ac	1091.061	1091.058 $[M+2H]^{2+}$
**27**	SV*c*[EDHFAKALGDTWLRIK]AA	FITC-Peg	1372.164	1372.152 $[M+2H]^{2+}$
**28**	SV*c*[EDHFAKALGDTWLRIK]RA	FITC-Peg	1414.688	1414.684 $[M+2H]^{2+}$
**29**	SV*c*[EAHFAAALGDTWLRIK]RR	FITC-Peg	1406.694	1406.692 $[M+2H]^{2+}$
