# Supplementary material for: A protein tertiary structure mimetic modulator of the Hippo signalling pathway
Source: Nat Commun. 2020 Oct 27;11:5425. doi: 10.1038/s41467-020-19224-8 (PMC7591920; doi:10.1038/s41467-020-19224-8)
Supplement: Supplementary file 3 — Reporting Summary [file 41467_2020_19224_MOESM3_ESM.pdf]

nature research

Corresponding author(s): TN Grossmann, H Waldmann

Last updated by author(s): Sep 23, 2020

Reporting Summary

Nature Research wishes to improve the reproducibility of the work that we publish. This form provides structure for consistency and transparency in reporting. For further information on Nature Research policies, see our Editorial Policies and the Editorial Policy Checklist.

Statistics

For all statistical analyses, confirm that the following items are present in the figure legend, table legend, main text, or Methods section.

n/a	Confirmed	
☐	☒	The exact sample size (*n*) for each experimental group/condition, given as a discrete number and unit of measurement
☐	☒	A statement on whether measurements were taken from distinct samples or whether the same sample was measured repeatedly
☐	☒	The statistical test(s) used AND whether they are one- or two-sided<br>*Only common tests should be described solely by name; describe more complex techniques in the Methods section.*
☐	☒	A description of all covariates tested
☐	☒	A description of any assumptions or corrections, such as tests of normality and adjustment for multiple comparisons
☐	☒	A full description of the statistical parameters including central tendency (e.g. means) or other basic estimates (e.g. regression coefficient) AND variation (e.g. standard deviation) or associated estimates of uncertainty (e.g. confidence intervals)
☐	☒	For null hypothesis testing, the test statistic (e.g. *F*, *t*, *r*) with confidence intervals, effect sizes, degrees of freedom and *P* value noted<br>*Give P values as exact values whenever suitable.*
☐	☒	For Bayesian analysis, information on the choice of priors and Markov chain Monte Carlo settings
☐	☒	For hierarchical and complex designs, identification of the appropriate level for tests and full reporting of outcomes
☐	☒	Estimates of effect sizes (e.g. Cohen's *d*, Pearson's *r*), indicating how they were calculated

Our web collection on statistics for biologists contains articles on many of the points above.

Software and code

Policy information about availability of computer code

Data collection	XDS 31-MAR-2018<br>XSCALE 31-MAR-2018<br>PHASER 2.5.6<br>PHENIX 1.9.1692
Data analysis	Pymol 2.4.0<br>MOE 2019.0102<br>GraphPad Prism v8.0.1<br>FlowJo version 10.1<br>CDNN 2.1<br>IncuCyte ZOOM 2016B<br>Genedata Screener 16.04

For manuscripts utilizing custom algorithms or software that are central to the research but not yet described in published literature, software must be made available to editors and reviewers. We strongly encourage code deposition in a community repository (e.g. GitHub). See the Nature Research guidelines for submitting code & software for further information.

Data

Policy information about availability of data

All manuscripts must include a data availability statement. This statement should provide the following information, where applicable:

- Accession codes, unique identifiers, or web links for publicly available datasets
- A list of figures that have associated raw data
- A description of any restrictions on data availability

Source data are provided with this paper. The materials and data reported in this study are available upon request from H.W. Crystal structure of the E4/mTEAD4 complex is available under PDB: 6SBA (http://dx.doi.org/10.2210/pdb6SBA/pdb). Crystal structure of the VGL4/mTEAD4 complex is available under PDB: 4NL0 (http://dx.doi.org/10.2210/pdb4LN0/pdb)

Field-specific reporting

Please select the one below that is the best fit for your research. If you are not sure, read the appropriate sections before making your selection.

- [x] Life sciences
- [ ] Behavioural & social sciences
- [ ] Ecological, evolutionary & environmental sciences

For a reference copy of the document with all sections, see nature.com/documents/nr-reporting-summary-flat.pdf

Life sciences study design

All studies must disclose on these points even when the disclosure is negative.

Sample size	Sample sizes were not predetermined using statistical analyse. All cell based assays were performed with the usual and sufficient sample size setting determined by previous experiments. All experiments reported have n number and repetitions reported.<br>in the figure legend. Experiments have been performed in three biological replicates, which consisted of at least two technical replicates. Statistical analysis was performed using Student's paired t-test ($p<0.05$ statistically significant)
Data exclusions	No data exclusions.
Replication	Results from all replicates in the given experiments were similar.<br>For wound closure assay: three biological replicates with three technical replicates<br>For proximity ligation assay: three biological replicates with two technical replicates<br>For in cell concentration assay: two biological replicates<br>For RT-qPCR assay: two biological replicates with three technical replicates<br>For Cell toxicity assessment: six biological replicates<br>For YAP localization (nucleus/cytosol) assay: three to four biological replicates<br>For High-content imaging-based analysis of cell cycle activity: six biological replicates<br>For Dose-dependent cell cycle stimulation assay: three biological replicates
Randomization	This is not relevant to this report because there is no source of bias in the data collection or the analysis. All samples were treated and assessed in the same manner and controls were used for the experiments.<br>same manner with the appropriate controls.
Blinding	Blinding is not relevant to this report. Data analysis was performed according to the number of the respective well and connected to the sample name later on.

Reporting for specific materials, systems and methods

We require information from authors about some types of materials, experimental systems and methods used in many studies. Here, indicate whether each material, system or method listed is relevant to your study. If you are not sure if a list item applies to your research, read the appropriate section before selecting a response.

Materials & experimental systems

n/a	Involved in the study	
[ ]	[x]	Antibodies
[ ]	[x]	Eukaryotic cell lines
[x]	[ ]	Palaeontology and archaeology
[ ]	[x]	Animals and other organisms
[x]	[ ]	Human research participants
[x]	[ ]	Clinical data
[x]	[ ]	Dual use research of concern

Methods

n/a	Involved in the study	
[x]	[ ]	ChIP-seq
[ ]	[x]	Flow cytometry
[x]	[ ]	MRI-based neuroimaging

Antibodies

Antibodies used	mouse anti-YAP, #sc-101199, dilution 1:100, Santa Cruz<br>rabbit anti-panTEAD, #13295, dilution 1:1000, Cell Signaling<br>mouse anti-αActinin, #A-7811, dilution 1:300, Sigma-Aldrich<br>rabbit anti-YAP, #14074, dilution 1:500, Cell Signalling<br>rabbit anti-Ki67, #ab16667, dilution 1:250, Abcam<br>donkey anti-rabbit PLUS, #DUO92002, dilution 1:5; Sigma-Aldrich<br>donkey anti-mouse MINUS, #DUO92004, dilution 1:5, Sigma-Aldrich<br>goat anti-mouse Alexa Fluor 568, #A-11004, dilution 1:1000, ThermoFisher Scientific<br>goat anti-rabbit AlexaFluor 488, #A-11008, dilution 1:1000, ThermoFisher Scientific
Validation	For rabbit anti-panTEAD (#13295) and rabbit anti-YAP (#14074) see: https://en.cellsignal.de/contents/our-approach/cst-antibody-validation-principles/ourapproach-validation-principles/<br>For mouse anti-αActinin (#A-7811) donkey anti-rabbit PLUS (#DUO92002), donkey anti-mouse MINUS (#DUO92004) see: https://origin-www.sigmaaldrich.com/life-science/cell-biology/antibodies/antibody-validation.html<br>For mouse anti-YAP, see: https://www.scbt.com<br>For rabbit anti-Ki67 (#ab16667) see: https://www.abcam.com/primary-antibodies/a-guide-to-antibody-validation<br>For goat anti-mouse Alexa Fluor 568 (#A-11004) and goat anti-rabbit AlexaFluor 488 (#A-11008) see: https://www.thermofisher.com/de/de/home/life-science/antibodies/invitrogen-antibody-validation.html

Eukaryotic cell lines

Policy information about cell lines

Cell line source(s)	RKO cells were purchased from ATCC, HeLa cells were purchased from DSMZ. ES cell line obtained from Wicell
Authentication	None of the cell lines were authenticated.
Mycoplasma contamination	All cell lines were tested negative for mycoplasma contamination.
Commonly misidentified lines (See ICLAC register)	No commonly misidentified cell lines were used.

Animals and other organisms

Policy information about studies involving animals; ARRIVE guidelines recommended for reporting animal research

Laboratory animals	Wistar rats were sacrificed on day 5-6 after birth (due to low age of rats, sex was not determined)
Wild animals	No experiment with wild animals is included in this report
Field-collected samples	No samples were collected in field
Ethics oversight	All experiments were approved by the official State animal care and use committee (LANUV, Recklinghausen, Germany AZ 84_02.04.2014.A333) The mice are housed under specific-pathogen-free conditions according to the guidelines of the Federation for Laboratory Animal Science Associations (FELASA). All experiments were conducted in accordance with the German federal law regarding the protection of animals and 'Guide for the Care and Use of Laboratory Animals' (National Institutes of Health publication 8th Edition, 2011).

Note that full information on the approval of the study protocol must also be provided in the manuscript.

Flow Cytometry

Plots

Confirm that:

- [x] The axis labels state the marker and fluorochrome used (e.g. CD4-FITC).
- [x] The axis scales are clearly visible. Include numbers along axes only for bottom left plot of group (a 'group' is an analysis of identical markers).
- [x] All plots are contour plots with outliers or pseudocolor plots.
- [x] A numerical value for number of cells or percentage (with statistics) is provided.

Methodology

Sample preparation	Using 12-well plates, HeLa cells (1*100.000cells/well) were plated and allowed to grow in growth medium for 24 h at 37 degrees under humidified atmosphere with 5% CO2. The cells were then treated with 5 uM and 15 uM final concentration of peptide in growth medium and a maximum of 0.5% DMSO. After 90 min of incubation at 37 degrees under humidified atmosphere with 5% CO2, the cells were washed with PBS three times. A solution of 0.05% trypsin and 0.02% EDTA in PBS

(PAN Biotech) was added and the cells were incubated for 5 min at at 37 degrees under humidified atmosphere with 5% CO2. Growth medium was used to stop the dissociation reaction and centrifugation (1.1 rcf, 3 min) allowed to harvest the cells. The cells were consecutively washed two times with PBS, suspended in 400 uL to 600 uL of PBS and transferred to a 5 mL Polysyrene round-bottom tube with cell-strainer cap (Falcon, Corning Inc.).

For flow cytometric analyses of P5-6 rat heart primary cells, 20.000 cells were seeded per well on a 0.1% gelatin- (Pan Biotech) coated 96 well plate (Sarstedt). Cells were incubated in IMDM media supplemented with 50 µg/mL penicillin/ streptomycin, 0.1 mM non-essential amino acids, 0.1 mM β-mercaptoethanol (all from Gibco) and 2 % fetal bovine serum (FBS, Pan Biotech) media at 37 °C and 5% CO2. One day after seeding the cells were manually treated once with the peptides of interest previously labeled with FITC at a final concentration of 5 µM. After 90 minutes treatment, cells were trypsinized (Trypsin 0.25%, Sigma) and thoroughly washed before peptide uptake was analyzed by FACS to avoid unspecific cell surface-binding. Peptide incorporation in live cells was measured as means of FITC fluorescence intensity.

Instrument	LSRII flow cytometer
Software	FlowJo version 10.1; source: Treestar
Cell population abundance	We analyse the peptide intake in the whole live population.
Gating strategy	Live cells are gated based in their size and granularity (SSC and FSC) to exclude cell debris, and peptide intake is measured as fluorescence intensity in the whole live population.

☒ Tick this box to confirm that a figure exemplifying the gating strategy is provided in the Supplementary Information.
